# Polymorphisms in monolignol biosynthetic genes are associated with biomass yield and agronomic traits in European maize (*Zea mays *L.)

**DOI:** 10.1186/1471-2229-10-12

**Published:** 2010-01-15

**Authors:** Yongsheng Chen, Imad Zein, Everton Alen Brenner, Jeppe Reitan Andersen, Mathias Landbeck, Milena Ouzunova, Thomas Lübberstedt

**Affiliations:** 1 Department of Agronomy, Iowa State University, Ames, Iowa 50011, USA; 2 Interdepartmental Genetics Graduate Program, Iowa State University, Ames, Iowa 50011, USA; 3 Department of Agronomy and Plant Breeding, Technical University of Munich, Am Hochanger 2, 85354 Freising-Weihenstephan, Germany; 4 Department of Genetics and Biotechnology, University of Aarhus, Research Center Flakkebjerg, 4200 Slagelse, Denmark; 5 KWS Saat AG, Grimsehlstr. 31,37555 Einbeck, Germany

## Abstract

**Background:**

Reduced lignin content leads to higher cell wall digestibility and, therefore, better forage quality and increased conversion of lignocellulosic biomass into ethanol. However, reduced lignin content might lead to weaker stalks, lodging, and reduced biomass yield. Genes encoding enzymes involved in cell wall lignification have been shown to influence both cell wall digestibility and yield traits.

**Results:**

In this study, associations between monolignol biosynthetic genes and plant height (PHT), days to silking (DTS), dry matter content (DMC), and dry matter yield (DMY) were identified by using a panel of 39 European elite maize lines. In total, 10 associations were detected between polymorphisms or tight linkage disequilibrium (LD) groups within the *COMT*, *CCoAOMT2*, *4CL1*, *4CL2*, *F5H*, and *PAL *genomic fragments, respectively, and the above mentioned traits. The phenotypic variation explained by these polymorphisms or tight LD groups ranged from 6% to 25.8% in our line collection. Only *4CL1 *and *F5H *were found to have polymorphisms associated with both yield and forage quality related characters. However, no pleiotropic polymorphisms affecting both digestibility of neutral detergent fiber (DNDF), and PHT or DMY were discovered, even under less stringent statistical conditions.

**Conclusion:**

Due to absence of pleiotropic polymorphisms affecting both forage yield and quality traits, identification of optimal monolignol biosynthetic gene haplotype(s) combining beneficial quantitative trait polymorphism (QTP) alleles for both quality and yield traits appears possible within monolignol biosynthetic genes. This is beneficial to maximize forage and bioethanol yield per unit land area.

## Background

Elevating the polysaccharide to lignin ratio is one possible approach to improve the quality of biofeedstocks for ethanol conversion [1]. It is believed that cell wall lignin content is negatively correlated with forage digestibility [2] and bioethanol production [3]. Removing lignin by oxidative pretreatment could significantly increase the release of available sugars in subsequent enzyme hydrolysis compared to the untreated control [4]. In maize, a 1% increase in available cellulose is expected to increase the potential ethanol production from 101.6 to 103.3 gallons per dry ton of biomass, as calculated using the U.S. Department of Energy's Theoretical Ethanol Yield Calculator and Feedstock Composition Database [5]. Theoretical maximum ethanol yields from biomass are highly correlated (r^2 ^= 0.9) with acid detergent lignin concentration [6]. According to Lorenz *et al*. [1], variation in ethanol yield is driven by glucan convertibility, which is highly correlated with ruminal digestibility and lignin content. Besides the lignin content, other aspects of cell wall lignification like the ratio of syringyl to guaiacyl lignin units affect cell wall digestibility [7,8] and, therefore, likely ethanol production from biofeedstocks. The syringyl to guaiacyl ratio impacts the efficiency of cell wall hydrolysis in forage sorghums [9]. In summary, modification of cell wall lignification is a promising route to improve the quality of bioenergy crops.

However, reduced lignin content can influence the overall plant performance. Generally, reduced lignin content results in weaker stalks, reduced stover and grain yield, and delayed maturity [10]. In maize, *brown-midrib *(*bm*) mutants show a decreased lignin content and increased cell wall digestibility [11]. For instance, lignin content is reduced by one third and cell wall digestibility is increased by 9% in *bm3 *lines or hybrids [12]. However, maize *bm *lines or hybrids show reduced vigor during vegetative growth, a high incidence of stalk breakage at maturity, and decreased grain and stover yield [13-16]. Similarly, *bm *hybrids of Sudan grass and sorghum also show reduced dry matter yield [17,18]. Genetically engineered tobacco with reduced *CCoAOMT *[19] or *PAL *activities [20], poplar with down-regulated *CCR *activity [21], *Arabidopsis *with a mutation in the *CCR1 *[22], *C3H *[23], and *C4H*genes [24], or with double mutations in the *COMT1*and *CCoAOMT1 *genes [25] showed reduced plant size. By silencing the *HCT *gene in *Arabidopsis*, Besseau *et al*. [26] obtained mutants with modified lignin structure as well as repressed plant growth. Silencing of *HCT *resulted in redirection of the metabolic flux into flavonoids, which suppressed auxin transport.

Decreased lignin content does not necessarily have negative effects on plant growth. After divergent selection for fiber concentration in maize, Wolf *et al*. [27] found only weak and inconsistent correlations between lignin content and various agronomic traits. Weller *et al*. [28] found no yield difference between *bm3 *and wildtype isolines. He *et al*. [29] developed *O*-methyltransferase down-regulated maize with a 17% decrease in lignin content, increased digestibility, without effect on dry matter yield. In aspen, repression of *4CL *led to a 45% reduction in lignin content [30]. While the structural integrity at both the cellular and whole-plant level was not affected, enhanced leaf, root, and stem growth were observed, as well as increased cellulose content [30]. By simultaneously silencing *HCT *and *CHS *genes, Besseau *et al*. [26] obtained normal growing *Arabidopsis *plants with substantially altered lignin composition. In summary, cell wall lignification is generally, but not always, negatively correlated with biomass yield and other agronomic traits. These correlations can be due to: (1) linkage of genes controlling monolignol biosynthesis and biomass yield, (2) pleiotropy at the level of genes but not QTPs within monolignol biosynthetic genes affecting both groups of traits, and (3) pleiotropic effects of QTP(s) within monolignol biosynthetic genes. The underlying genetic cause(s) for these correlations impact the strategy for breeding of bioenergy crops.

Ten enzymes are involved in converting phenylalanine to monolignols in maize, and the majority is encoded by two or more genes [31]. Four genes encode PAL proteins in *Arabidopsis*, which catalyze the first step in the phenylpropanoid pathway [32]. In maize, PAL has both phenylalanine and tyrosine ammonia lyase activity [33] and at least five contigs with *PAL*/*TAL *annotation were identified [31]. The other enzymes involved in biosynthesis of monomers include cinnamate 4-hydroxylase (C4H), 4-coumarate:CoA ligase (4CL), hydroxycinnamoyl-CoA transferase (HCT), *p*-coumarate 3-hydroxylase (C3H), caffeoyl-CoA *O*-methyltransferase (CCoAOMT), cinnamoyl-CoA reductase (CCR), ferulate 5-hydroxylase (*F5H*), caffeic acid *O*-methyltransferase (COMT), and cinnamyl alcohol dehydrogenase (CAD), with at least two, seven, two, one, five, eight, two, one, and seven sequences were identified, respectively [31]. Association mapping is a promising approach to identify candidate QTPs for traits of interest [34-37]. The *CCoAOMT2 *gene is co-localized with a QTL for cell wall digestibility and lignin content [38], and an 18-bp indel in the first exon was found to be associated with cell wall digestibility [34]. In addition, associations have been identified between neutral detergent fiber (NDF) and polymorphisms within *PAL*, *4CL1*, *C3H*, and *F5H *genes, between *in vitro *digestibility of organic matter (IVDOM) and polymorphisms within *PAL*, *4CL1*, and *C3H*, and between digestibility of neutral detergent fiber (DNDF) and polymorphisms in *C3H *and *F5H *genes [35,36]. However, genes encoding any of these 10 enzymes have so far not been studied in relation to biomass yield-related traits. In this study, the relationship between 10 monolignol biosynthetic genes belonging to eight enzyme encoding genes or gene families and the biomass yield-related traits: plant height (PHT), days to silking (DTS), dry matter content (DMC), and dry matter yield (DMY) were analyzed. Only one or two gene member(s) of each gene family were amplified. Our objectives were to investigate, (1) whether candidate quantitative trait polymorphisms (QTPs) for these four traits can be identified in monolignol biosynthetic genes, and (2) whether candidate QTPs for biomass yield-related traits and cell wall digestibility traits act pleiotropically by comparing the results of this study with results from previous forage trait association studies [[35,36], Brenner *et al*.: Polymorphisms in *O*-methyltransferase genes are associated with stover cell wall digestibility in European maize (*Zea mays *L.), submitted]. The results are discussed with respect to implications for breeding of maize for forage and lignocellulosic ethanol production.

## Results

### Phenotypic data analyses

Mean phenotypic values for individual lines across four environments ranged from 109.3 to 197.1 cm for PHT, 68.5 to 85.7 days for DTS, 23.2% to 36.0% for DMC, and 2.5 to 8.4 t/ha for DMY. Overall mean values were 152.0 cm, 78.6 days, 28.5%, and 5.3 t/ha, respectively, for these four traits (Table 1). Variance components for genotype and interactions between genotype and environment were significant (P = 0.01) and variance components for environment were significant (P = 0.01) for PHT, DTS, and DMC. Heritabilities were 88.0%, 92.0%, 85.7%, and 81.9% for PHT, DTS, DMC, and DMY, respectively (Table 1). Means of dent lines were significantly higher than means of flint lines for DTS (P = 0.01), DMY (P = 0.01), and PHT (P = 0.05), whereas DMC was not significantly different between dent and flint lines.

**Table 1:** Phenotypic means, variance components, and heritabilities for four agronomic traits across four environments.

Line	PHT	DTS	DMC	DMY
F_AS1	109.3	70.8	36.0	3.2
F_AS2	154.4	75.3	27.0	3.9
F_AS3	136.4	76.3	25.4	5.6
F_AS4	150.7	71.7	25.5	4.8
F_AS5	121.2	71.5	28.8	2.8
F_AS6	151.7	78.3	29.4	4.4
F_AS7	141.1	74.0	27.5	4.2
D_AS8	137.7	74.5	29.9	4.0
D_AS9	179.6	84.5	27.4	8.1
D_AS10	168.8	85.3	29.0	7.4
D_AS11	159.7	82.5	25.4	5.5
F_AS12	145.2	80.3	30.4	3.7
F_AS13	150.0	80.7	30.7	4.2
F_AS14	171.4	79.2	23.2	5.4
F_AS15	152.4	77.5	29.5	4.1
F_AS16	128.2	72.5	26.9	4.2
F_AS17	197.1	77.5	30.7	5.7
F_AS18	122.6	68.5	32.7	2.5
F_AS19	159.9	75.8	28.0	4.7
F_AS20	164.2	77.8	25.9	5.9
F_AS21	151.4	76.2	26.9	5.0
F_AS22	144.4	78.0	28.9	6.9
F_AS23	141.1	77.5	27.0	5.8
F_AS24	133.6	75.8	24.7	4.8
D_AS25	150.1	80.0	30.0	4.3
D_AS26	146.0	81.0	27.9	6.2
D_AS27	146.5	83.7	26.6	5.4
D_AS28	158.7	81.2	29.0	4.5
D_AS29	155.8	80.2	26.0	6.7
D_AS30	168.7	79.3	34.3	5.1
D_AS31	161.2	81.3	31.3	5.0
D_AS32	167.2	83.5	27.6	6.4
D_AS33	177.8	81.3	28.0	8.4
D_AS35	155.0	81.7	26.2	7.2
D_AS36	171.8	85.7	28.5	7.5
D_AS37	157.1	81.2	31.3	5.3
F_AS38	154.2	80.8	30.1	6.0
D_AS39	131.0	80.5	29.5	5.3
F_AS40	153.7	80.2	29.8	5.9
				
Phenotypic means				
Flint	147.0 ± 18.5	76.2 ± 3.4	28.4 ± 2.9	4.7 ± 1.1
Dent	158.4 ± 13.4	81.6 ± 2.6	28.7 ± 2.3	6.0 ± 1.4
Overall	152.0 ± 17.3	78.6 ± 4.1	28.5 ± 2.6	5.3 ± 1.4
				
Variance components				
Lines	8.3**	12.6**	7.00**	5.5**
Environments	44.0**	133.3**	30.3**	2.5
L×E	3.5**	6.2**	2.7**	4.0**
LSD5	16.8	3.3	2.7	1.64
				
Heritability% and CI interval				
Heritability%	88.0	92.0	85.7	81.9
90% CI on *h*^2^	78.9-92.6	85.7-95.3	74.9-91.3	68.2-88.9

These lines are the same 40 lines (except D_AS34) used by Andersen *et al*.[36].

Flint- and dent lines are denoted by F_ and D_ prefixes, respectively.

PHT: plant height (cm); DTS: days to silking; DMC: dry matter content of stover; DMY: dry matter yield of stover (tons per hectare)

LSD5: least significant difference at 5% level between lines

CI: confidence interval

*, ** significant at 5% and 1% level, respectively.

PHT was positively correlated with DTS and DMY at both phenotypic and genotypic levels, with phenotypic and genotypic correlation coefficients ranging from r = 0.62 (P = 0.01) to 0.69 (P = 0.01). DNDF was negatively correlated with PHT (phenotypic correlation coefficient r_p _= -0.45, genotypic correlation coefficient r_g _= -0.47, P = 0.01), as well as DMY (r_g _= -0.24, P = 0.05) (Table 2).

**Table 2:** Phenotypic and genotypic (*italics*) correlations between forage quality and yield-related traits.

	DTS	DMC	DMY	WSC	IVDOM	NDF	DNDF
PHT	0.64**	-0.17	0.64**	0.24	-0.31	0.06	-0.45**
	0.62**	*-0.17*	0.69**	0.24*	-0.35**	*0.11*	-0.47**
DTS		-0.13	0.72**	0.17	-0.17	0.08	-0.26
		*-0.09*	0.78**	*0.14*	-0.22*	*0.17*	-0.28*
DMC			-0.35*	-0.51**	-0.39*	0.61**	-0.15
			-0.37**	-0.56**	-0.40**	0.66**	-0.17*
DMY				0.44**	0.00	-0.23	-0.22
				0.47**	*-0.03*	-0.23*	-0.24*

** Significant at P = 0.01. * Significant at P = 0.05.

PHT: plant height in cm

DTS: days from sowing to silking

DMC: % dry matter content of stover

DMY: Dry matter yield of stover in tons per hectare

WSC: Water soluble carbohydrates

IVDOM: *In vitro *digestibility of organic matter

NDF: Neutral detergent fiber

DNDF: Digestibility of neutral detergent fiber

Previous studies reported the haplotype diversity of these ten monolignol biosynthetic genes [[35,36,39], Brenner *et al*.: Polymorphisms in *O*-methyltransferase genes are associated with stover cell wall digestibility in European maize (*Zea mays *L.), submitted]. The number of haplotypes ranged from 2 to 12 for the ten monolignol biosynthetic genes (see Additional file 1). *COMT*, *CCoAOMT1*, and *F5H *showed the largest phenotypic ranges among haplotype classes for PHT (121.2-171.4 cm) and DMC (23.2%-30.3%), DMY (4.1-8.4 t/ha), and DTS (72.2-84.1 days), respectively.

### Association analyses

Association analyses revealed that six genes, coding for COMT, CCoAOMT2, 4CL1, 4CL2, F5H, and PAL proteins, were associated with at least one of the four biomass yield-related traits. 10 associations were identified by GLM when including population structure in the analysis and controlling for multiple testing. Among those, seven were validated by MLM (Tables 3 and 4), which, in addition to population structure, corrects for finer scale relative kinship. However, none of these polymorphisms identified by MLM remained significant after controlling for multiple testing by FDR. At the *PAL *locus a tight LD group containing 17 polymorphisms with *r*^2 ^= 1 was associated with days to silking (DTS). The 39 lines were classified into two groups by this LD group. The lines including AS1-8, 11-22, 24, and 29 were six days earlier than the remaining lines. This LD group explained 7% of the total DTS variation in our population. At the *4CL2 *locus, a tight LD group consisting of two SNPs (at position 192 and 217) in complete LD explained 14.3% of the phenotypic variation for PHT. The SNP at position 217 led to an amino acid change. The lines with the TG allele at these two positions were on average 17 cm higher than the lines with the CA allele. At the *CCoAOMT2 *locus, three polymorphisms (an indel starting at position 75, two SNPs at position 144 and 406) were in a tight LD group with *r*^2 ^> 0.89, which explained 23.5% of the phenotypic variation for DMC. Another indel, which starts at position 663 in this locus explained 25.8%, 18.5%, and 10.5% of variation for PHT, DTS, and DMC, respectively. At the *4CL1 *locus, two indels (starting at position 454 and 810) were both associated with DTS, and explained 20.2% and 6% of the phenotypic variation, respectively. These two indels both resulted in reading frame shift, with one of those being a singleton. Lines with an Adenine insertion at position 454 silked on average three days earlier than the remaining lines. The *COMT *gene has been shown to strongly affect cell wall digestibility and plant height. However, only one polymorphism was detected for associations with DTS. The indel in the 3'UTR was detected only by GLM and explained 10.3% of the phenotypic variation for DTS. Finally, one trait association was detected at the *F5H *locus, which was a missense substitution at position 65 and explained 22.4% of the phenotypic variation for DTS.

**Table 3:** Associations between individual polymorphisms or LD groups and biomass yield and agronomic traits.

Gene	Position	Associated trait	R^2%	Identified by
*PAL*	947-1655^LD,1^	DTS	7.4%	GLM*, MLM**
*4CL2*	192, 217^LD, 2^	PHT	14.3%	GLM*, MLM*
*COMT*	2358	DTS	10.3%	GLM*
*F5H*	65	DTS	22.4%	GLM**
*4CL1*	454	DTS	20.2%	GLM*, MLM*
	810	DTS	6.0%	GLM*, MLM**
*CCoAOMT2*	75,144, 406^LD,3^	DMC	23.5%	GLM*, MLM*
	663	PHT	25.8%	GLM*
	663	DTS	18.5%	GLM*, MLM**
	663	DMC	10.5%	GLM*, MLM**

Positions of trait associated polymorphisms are presented according to public reference sequences.

^LD ^linkage group in a linkage block; ^1 ^the LD group contains 17 polymorphisms in complete LD in *PAL *and 947 and 1655 are the first and last polymorphisms; ^2 ^the LD group contains two polymorphisms which are in complete LD; ^3 ^the LD group contains three polymorphisms with *r*^2 ^> 0.89. Associations identified by GLM were after controlling multiple testing, while those identified by MLM were not. Polymorphism positions are denoted by the position in our alignment; *R*^2^% is the proportion of phenotypic variance explained by the detected polymorphisms/LD group; * and ** mean P < 0.05 and P < 0.01, respectively.

*4CL*: 4-coumarate:CoA ligase, *C3H*: p-coumarate 3-hydroxylase, *C4H*: cinnamate 4-hydroxylase, *CAD*: cinnamyl alcohol dehydrogenase, *CCoAOMT*: caffeoyl-CoA O-methyltransferase, *COMT*: caffeic acid O-methyltransferase, *F5H*: ferulate 5-hydroxylase; PHT: plant height in cm; DTS: days from sowing to silking; DMC: % dry matter content of stover; DMY: dry matter yield of stover in tons per hectare

**Table 4:** Polymorphism character and position in reference sequence.

Gene	Position	Polymorphism	Position in reference sequence
*PAL*	947	Intron SNP	TTTAGAGACGATCGCAATCCA(C/T)
	1652,1655	Intron SNP	CCTTGTGCGCTGGCTCCC(T/C)TT(C/T)
*4CL2*	192	synonymous SNP	TACTGCTTCGGGAAGATGGG(T/C)
	217	Val to Ile SNP	TGGCGGAGCGGGCGTGCCTG(G/A)
*COMT*	2358	3'UTR indel	CGCCGTCGTCGTCTTCTTCT(+/-)
*F5H*	65	Leu to Pro SNP	ACGCGCGACAATATCAAGGC(T/C)
*4CL1*	454	Exon indel	TTCGTCGGCAAGGTGCGGGGG(+/-)
	810^s^	Exon indel	GCTGTGCGGGATGCGCGCCGG(+/-)
*CCoAOMT2*	75	Exon Indel	CAGGCCAACGGCAACGGCAA(+/-)
	144	synonymous SNP	CTGCTCAAGAGCGACGACCT(C/G)
	406	Intron SNP	ACCGAGATCTGAGAACGAAC(A/G)
	663	Intron indel	CCTAGGATCTTAACCACCCC(+/-)

Polymorphism character and position in reference sequence. (N/N): SNP substitution; (+/-): indel; s: singleton; M73235, AY323238, AY279014, AX204867, AX204868, and AX204869 were used as reference sequences for *COMT*, *CCoAOMT1*, *CCoAOMT2*, *4CL1*, *4CL2*, and *F5H *respectively. The reference sequences for *PAL *are the conserved sequences before each polymorphism in our alignment.

### Pleiotropic polymorphisms affecting biomass yield and forage quality

In order to increase the chance of finding potential pleiotropic QTP affecting both biomass yield-related and digestibility traits, associations of monolignol biosynthetic genes [[35,36], Brenner *et al*.: Polymorphisms in *O*-methyltransferase genes are associated with stover cell wall digestibility in European maize (*Zea mays *L.), submitted] were determined without multiple test adjustment. In our study, two additional trait associations were detected only by MLM, one of which was an association between a synonymous SNP in the *COMT *gene and PHT, the other one was between a tight LD group in the *F5H *gene (two SNPs at position 5 and 6 in complete LD) and DMY. Despite of these relaxed statistical test conditions, only two polymorphisms in 10 monolignol biosynthetic genes were associated with both biomass yield-related and cell wall digestibility traits. The indel starting at position 810, resulting in a reading frame shift in the *4CL1 *gene, was associated with IVDOM [36] and DTS identified by both GLM and MLM. It was also associated with NDF identified by GLM [36]. The tight LD group with two SNPs in complete LD in the *F5H *gene, resulting in a substitution from Proline to Arginine, was associated with both DMY (by MLM) and NDF (by GLM) [36]. In addition, the tight LD group in the *PAL *gene showing association with DTS in our study was also associated with NDF [35]. However, the association between this LD group and NDF was only detected when population structure was not considered. In summary, no pleiotropic polymorphisms associated with DNDF and DMY or PHT were identified.

## Discussion and conclusion

### Impact of the association analysis method on QTP identification

Two statistical approaches (GLM and MLM) were employed as in previous association studies for better comparison across quality [[35,36], Brenner *et al*.: Polymorphisms in *O*-methyltransferase genes are associated with stover cell wall digestibility in European maize (*Zea mays *L.), submitted] and yield-related traits (this study). In those former studies, the same line panel, gene sequences, and marker data have been used. Inclusion of both population structure and relative kinship reduces the number of false positive associations compared to including population structure alone [40]. In the present study, most of the associations identified for biomass yield and other agronomic traits by GLM were also identified by MLM, although none of the associations identified by MLM remained significant after controlling for multiple testing. Therefore, we can not exclude the possibility that familiar relatedness resulted in false positives. However, this result might also suggest that inclusion of relative kinship information might in some cases mask genuine associations, comparable to likely false negatives of flowering time caused by inclusion of population structure for the *Dwarf8 *gene in European maize [41]. In this example, likely true effects of QTP on flowering time were confounded with presence of one particular allele set in flint, the other in dent lines.

### Characterization of polymorphisms associated with biomass yield and agronomic traits

We compared trait-associated (27) with not-associated polymorphisms (255) within the 10 monolignol biosynthetic genes regarding (i) the distribution among SNPs and indels, and (ii) polymorphisms among coding and non-coding sequences. Based on Chi-square tests, trait-associated polymorphisms for biomass yield-related traits were not preferentially due to either SNPs or indels, and not primarily located in either coding or non-coding gene regions.

Polymorphisms in conserved motifs with impact on protein function or abundance are more likely candidates for causative QTPs [42]. Within the *PAL *gene in our study, 1 out of 17 polymorphisms in the LD group associated with DTS was located within a possible bipartite RAV1 binding site [43,44]. RAV1 has been suggested as a negative regulator of plant growth and development [45]. In addition, five polymorphisms in the same LD group were located within Dof-like motifs [43]. Dof transcription factors play a critical role in plant growth and development [46]. Those six polymorphisms are more likely candidates for causative QTPs, whereas the remaining 11 significant associations within the same LD group are more likely due to linkage. To pinpoint causative polymorphisms, further dissection based on additional alleles at low LD is required. In the *CCoAOMT2 *gene, a 40-60 bp indel at position 663 was just six base pairs upstream of a 3' splicing donor site, spanning a potential "branching site" for splicing. Consequently, this indel might affect splicing and in this way interfere with the mRNA sequence and function of *CCoAOMT2*. Moreover, this indel also spanned part of a bipartite RAV1 binding site [43]. Interestingly, this site was associated with three biomass yield-traits. Although LD decay was rapid in *CCoAOMT2*, the indel and two SNPs, which are at positions 75, 144, and 406, respectively, were tightly linked (*r*^2 ^> 0.89). The indel resulted in two amino acid (Asparagine and Glycine) deletions compared with the *CCoAOMT2 *allele of maize inbred line F2 (NCBI accession number AY279014.1). The other two SNPs were either synonymous or intron located SNPs. Thus, the indel is a more promising candidate QTP compared to the other two SNPs. Two DTS associated polymorphisms in *4CL1*, which were both single nucleotide indels, led to frame shift mutations. One indel starting at position 810 introduced a premature stop [36]. The other indel changed the peptide sequence substantially, since it is located close to the transcription initiation site. In *4CL2*, two polymorphisms in complete LD were associated with PHT. One of them changed the amino acid sequence and is, therefore, a more likely candidate QTP. In the *F5H *gene, Leucine to Proline and Proline to Arginine substitutions, were associated with DTS and DMY, respectively. Both are expected to change protein structure dramatically based on the Blosum-62 substitution matrix [47]. Proline is very different from other amino acids due to its aliphatic side chain bonded to both nitrogen and *α*-carbon atoms. In summary, some of the above mentioned trait associated polymorphisms or LD groups likely change protein sequence and expression dramatically, and are consequently the most likely QTPs affecting agronomic traits. However, future studies with maize populations with very low LD or alternative approaches are required for validation.

### Pleiotropic effects of monolignol biosynthetic genes

Besides biosynthesis of lignin monomers, the monolignol biosynthetic pathway is involved in biosynthesis of salicylates, coumarins, hydroxycinnamic amides, pigments, UV light protectants, antioxidants, and flavonoids [48]. Jone [49] concluded that phenylpropanoid compounds are involved in controlling plant development, growth, xylogenesis, and flowering. For example, chalcone and naringenin, two intermediates in the phenylpropanoid metabolism in plants, inhibit *4CL *activity [50] and suppress the growth of at least 20 annual plant species including maize [51]. Moreover, mutants in genes coding for C3H, C4H, PAL, CCoAOMT1, CCR1 and HCT show effects on plant growth [19-26]. This is likely due to redirection of metabolic flux and accumulation of compounds, like naringenin, flavonoids, chalcone, which have the potential to perturb hormone homeostasis and ultimately affect plant growth.

In our study, polymorphisms affecting both biomass yield and cell wall digestibility were identified in six monolignol biosynthetic genes (encoding for COMT, CCoAOMT2, 4CL1, 4CL2, F5H, and PAL). These findings indicate that at least some of the monolignol biosynthetic genes act pleiotropically on both lignin content or composition and biomass yield or other agronomic traits. However, only two polymorphisms, the indel at position 810 in the *4CL1 *gene and the LD group with SNPs resulting in substitution from Proline to Arginine [36] in the *F5H *gene, were found to be associated with both biomass yield and cell wall digestibility traits without controlling multiple testing. After controlling multiple testing, only the indel in the *4CL1 *gene was associated with both DTS and IVDOM. Thus, the majority of QTPs identified in our study affected only one of the two groups of traits. Intragenic linkage of respective QTPs was more abundant than pleiotropic QTPs. According to our findings, most QTPs for both groups of traits are expected to segregate independently in germplasms with low LD.

Another important implication from our results is, that pleiotropy identified by comparison of wild-type with knock-out alleles, might in several cases turn out to be due to close linkage of intragenic QTPs with effects on different pathways and traits. An example is the well-studied *Dwarf8 *gene. This gene has been shown to affect plant height, when comparing mutant and wild type alleles [52]. However, association analyses with a range of wildtype alleles revealed candidate QTPs for flowering time, but not for plant height [37]. In *Dwarf8*, the DELLA domain is thought to affect plant height [52], while other polymorphisms affect flowering time. The DELLA domain was conserved in the 92 inbred lines used for an association analysis [37]. Similarly, previous *bm3 *mutant studies implied that the *COMT *coding gene acts pleiotropically on both forage quality and yield characters. However, after adjustment for multiple testing only one polymorphism was associated with DTS in our analysis, whereas eight different polymorphisms were associated with DNDF [Brenner *et al*.: Polymorphisms in *O*-methyltransferase genes are associated with stover cell wall digestibility in European maize (*Zea mays *L.), submitted]. Since earlier reports on pleiotropy of *bm *mutations were based on isogenic lines, another explanation might be closely linked genes in introgressed donor segments affecting either quality or yield characters.

### Implications for plant breeding

Although the genetic correlation between DNDF and DMY was significant (P = 0.05), it was very low (r = -0.24) in these 39 inbred lines. Hence, it is very likely that the majority of genes affecting either biomass yield or cell wall digestibility traits are different. Our results support that monolignol biosynthetic genes affect both biomass yield-related and cell wall digestibility traits. Intragenic linkage of QTPs was the more frequent cause for "pleiotropy" compared to pleiotropic polymorphisms. No QTP in our study was associated with PHT and DNDF, or DMY and DNDF. Considering these correlations and association data together, we conclude that breeders can employ optimal wildtype alleles for monolignol biosynthetic genes to improve cell wall digestibility, without penalty on DMY.

## Methods

### Plant materials

A panel of 39 European elite inbred lines including 22 Flint and 17 Dent lines used for forage quality studies [[35,36], Brenner *et al*.: Polymorphisms in *O*-methyltransferase genes are associated with stover cell wall digestibility in European maize (*Zea mays *L.), submitted] were employed in this study. Five lines (AS01 = F7, AS02 = F2, AS03 = EP1, AS39 = F288, and AS40 = F4) were from the public domain, the remaining inbred lines were provided by KWS Saat AG (Table 1). These lines were selected as extremes with respect to DNDF from a larger set of > 300 European lines (unpublished data).

### Agronomic trials

Four biomass yield-related traits were evaluated for these 39 lines in Grucking (sandy loam) and Bernburg (sandy loam) in 2002 and 2003, respectively. Field trials were performed as 7*7 lattice design with two replications in each environment. 20 plants were planted per plot in single row plots, 3 m long and 0.75 m apart. Analysis of forage quality related traits: water soluble carbohydrate (WSC), *in vitro *digestibility of organic matter (IVDOM), neutral detergent fiber (DNF), and digestibility of neutral detergent fiber (DNDF) have previously been reported [36]. In our study, four biomass yield-related traits PHT, DTS, DMC, and DMY were analyzed. PHT was measured as distance from soil level to the lowest tassel branch after flowering. DTS was measured as days from sowing to silking. Dry matter content (DMC) of stover (g/kg) (ears were manually removed) was determined 50 days after flowering and dry matter yield (DMY) was measured in tons per hectare.

### Phenotypic data analyses

Mean values, heritability, and variance components of each biomass yield-related trait and correlations between the above mentioned eight traits were calculated in PLABSTAT version 3A [53]. Briefly, analyses of variance were performed for each experiment separately. Adjusted entry means and effective error mean squares were used to compute the combined variances and covariances across environments for each trait. The sums of squares for entries were subdivided into variation among inbred lines, environments, interaction between inbred lines and environments, and error. Variance components were computed for lines and environments, considering them as random effects in the statistical model: Phenotype = effects of lines + effects of environments + effects of lines by environment (P = mean+L+E+L×E). *F-*tests were employed for testing the homogeneity of lines, environments and interactions between lines and environments according to the approximation given by Satterthwaite [54]. Heritabilities (*h*^2^) for each trait were calculated on an entry-mean basis, and confidence intervals for *h *^2^were obtained according to Knapp *et al*. [55]. Phenotypic and genotypic correlations between eight traits were calculated by standard procedures [56].

### DNA extraction, amplification, and sequencing

Leaves of each of the 39 lines were harvested in the greenhouse three weeks after germination for DNA extraction by the Maxi CTAB method [57]. Primers for PCR amplification of *C4H*, *4CL1*, *4CL2*, *C3H*, *F5H*, *CAD*, *PAL*, *COMT*, *CCoAMT1*, and *CCoAMT2*, as well as amplification conditions were described elsewhere [[35,36,39], Brenner *et al*.: Polymorphisms in *O*-methyltransferase genes are associated with stover cell wall digestibility in European maize (*Zea mays *L.), submitted]. Two overlapping fragments were amplified for *PAL *and *COMT *to cover the complete genes, whereas partial gene sequences were obtained for the other genes. Sequences were aligned in CLUSTALW [58] and stored in Nexus format for haplotype analysis in DnaSP [59]. Only one member of the *PAL*, *C4H*, *F5H*, and *CAD *gene families, respectively, was amplified. The reference sequences used for primer design were L77912, AY104175, AX204869, and AJ005702 (GenBank accession number). Primers for *COMT *and *C3H *were designed based on M73235 and AY17051. Two members of the *CCoAOMT *gene family corresponding to *CCoAOMT1 *and *CCoAOMT2 *[60] were amplified. Two members of the *4CL *gene family corresponding to the sequences reported by Puigdomenech *et al*. [61] were amplified.

### Population structure and association analysis

101 publicly available simple sequence repeat markers (SSR) http://www.maizegdb.org/ssr.php, evenly distributed across the whole genome of maize, were employed to genotype the 39 inbred lines. SSR data were used to infer the population structure in Structure 2.0 software [62,63]. Individual lines were grouped based on marker profiles by the Bayesian clustering method of Structure 2.0. The membership coefficients for each individual in each subpopulation were calculated with a burn-in length of 50,000 followed by 50,000 iterations and stored in a Q matrix. Inbreds were treated as haploids. Based on these SSR marker data, finer scale relative kinship (K)- Loiselle kinship coefficients [64] between lines were calculated in SPAGeDi [65]. Values on the diagonal of the K matrix were set as 2, and negative values in the matrix indicating that two individuals were less related than randomly chosen individuals [65] were set to 0.

Association analyses were carried out using the general linear model (GLM), and mixed linear model (MLM) in TASSEL 2.01 software [41] to test associations between polymorphisms of the 10 monolignol biosynthetic genes and four biomass traits. The threshold for *P*-values was set to 0.05. In all models, the Q matrix was used to account for overall population structure. 10,000 permutations were used to determine the *P*-value for association of each polymorphism by GLM. The *P*-value adjusted for multiple tests was obtained by a step-down MinP procedure [66], implemented in TASSEL. For MLM, the K matrix was included to account for relative kinship between individuals [41]. Trait associated polymorphisms with *r*^2 ^> 0.85 and *D*' > 0.9 were assigned to a tight LD group [67]. The phenotypic variation explained by this tight LD group was considered to be equal to the phenotypic variation of that polymorphism with the largest effect in this region. The False Discovery Rate (FDR) was determined to correct for multiple testing by MLM [68].

## Abbreviations

4CL: 4-coumarate:CoA ligase; C3H: p-coumarate 3-hydroxylase; C4H: cinnamate 4-hydroxylase; CAD: cinnamyl alcohol dehydrogenase; CCoAOMT: caffeoyl-CoA O-methyltransferase; COMT: caffeic acid O-methyltransferase; F5H: ferulate 5-hydroxylase; PAL: phenylalanine ammonia-lyase; indel: insertion-deletion polymorphism; LD: linkage disequilibrium; QTP: quantitative trait polymorphism; SNP: single-nucleotide polymorphism; IVDOM: *in vitro *digestibility of organic matter; DNDF: digestibility of neutral detergent fiber; NDF: neutral detergent fiber; WSC: water soluble carbohydrates; PHT: plant height; DTS: days to silking; DMC: dry matter content; DMY: dry matter yield

## Authors' contributions

YC performed the data analysis and prepared the manuscript. IZ carried out allele sequencing. EAB was involved in data analysis. JRA helped with statistical data analysis. MO provided the SSR data. ML has run the field trial. TL coordinated the project and together with YC prepared the manuscript. All authors read and approved the final manuscript.

## Supplementary Material

Additional file 1 **Supplementary table**. Haplotype number, average, minimum, and maximum of biomass yield-related trait values for each monolignol biosynthetic gene. Haplotype numbers and inbred lines included in each haplotype group: see [35] for *PAL*, [36] for *4CL2*, *4CL1*, *CAD*, *C3H*, *C4H*, and *F5H*, and [Brenner *et al*.: Polymorphisms in *O*-methyltransferase genes are associated with stover cell wall digestibility in European maize (*Zea mays *L.), submitted] for *CCoAOMT1*, *CCoAOMT2*, *COMT*. Click here for file
