# Supplementary material for: Polymorphisms in monolignol biosynthetic genes are associated with biomass yield and agronomic traits in European maize (Zea mays L.)
Source: BMC Plant Biol. 2010 Jan 15;10:12. doi: 10.1186/1471-2229-10-12 (PMC2827421; doi:10.1186/1471-2229-10-12)
Supplement: Additional file 1 — Supplementary table. Haplotype number, average, minimum, and maximum of biomass yield-related trait values for each monolignol biosynthetic gene. Haplotype numbers and inbred lines included in each haplotype group: see [35] for PAL, [36] for 4CL2, 4CL1, CAD, C3H, C4H, and F5H, and [Brenner et al.: Polymorphisms in O-methyltransferase genes are associated with stover cell wall digestibility in European maize (Zea mays L.), submitted] for CCoAOMT1, CCoAOMT2, COMT. [file 1471-2229-10-12-S1.DOC]

**Additional file 1**

Haplotype number, average, minimum, and maximum of biomass yield-related trait values for each monolignol biosynthetic gene**.** Haplotype numbers and inbred lines included in each haplotype group: see [35] for *PAL*, [36] for *4CL2*, *4CL1*, *CAD*, *C3H*, *C4H*, and *F5H*, and [39] for *CCoAOMT1*, *CCoAOMT2*, *COMT*.

|  |  | PHT | | | DTS | | | DMC | | | DMY | | |
| --- | --- | --- | --- | --- | --- | --- | --- | --- | --- | --- | --- | --- | --- |
| Gene | Hap Num | Hap Min | Hap Max | Mean | Hap Min | Hap Max | Mean | Hap Min | Hap Max | Mean | Hap Min | Hap Max | Mean |
| *COMT* | 12 | 121.2 | 171.4 | 146.9 | 71.5 | 82.4 | 78.1 | 23.2 | 30.3 | 28.6 | 2.7 | 6.0 | 4.9 |
| *CCoAOMT1* | 9 | 131.0 | 177.8 | 154.4 | 75.7 | 85.8 | 79.7 | 26.0 | 29.9 | 28.4 | 4.1 | 8.4 | 5.9 |
| *CCoAOMT2* | 6 | 131.0 | 161.2 | 147.9 | 74.0 | 81.3 | 79.1 | 27.6 | 31.2 | 29.8 | 4.2 | 5.9 | 5.0 |
| *PAL* | 8 | 146.5 | 179.6 | 156.5 | 75.7 | 84.6 | 80.5 | 26.5 | 31.2 | 28.6 | 4.2 | 8.1 | 5.6 |
| *C4H* | 4 | 143.9 | 164.1 | 152.1 | 75.5 | 80.0 | 78.3 | 28.2 | 28.8 | 28.6 | 4.5 | 5.6 | 5.1 |
| *4CL1* | 4 | 152.5 | 167.2 | 158.4 | 76.2 | 83.6 | 4.5 | 27.6 | 31.2 | 3.8 | 4.9 | 6.4 | 5.4 |
| *4CL2* | 7 | 142.0 | 162.4 | 150.2 | 75.0 | 80.6 | 78.4 | 26.9 | 30.6 | 28.7 | 3.9 | 6.0 | 4.9 |
| *C3H* | 7 | 150.7 | 171.4 | 159.9 | 71.7 | 81.1 | 79.0 | 23.2 | 29.5 | 26.8 | 4.5 | 5.4 | 5.1 |
| *CAD* | 2 | 152.2 | 155.3 | 153.7 | 75.3 | 79.1 | 77.2 | 28.3 | 28.5 | 28.4 | 4.9 | 5.3 | 5.1 |
| *F5H* | 5 | 133.6 | 169.4 | 150.7 | 72.2 | 84.1 | 77.7 | 24.7 | 29.2 | 27.5 | 3.8 | 6.2 | 5.1 |
